# Intractable Sneezing Unfolding a Hideous Truth

**DOI:** 10.7759/cureus.15268

**Published:** 2021-05-27

**Authors:** Jia Ji Ng, Salina Husain, Hui Yan Ong, Farah Dayana Zahedi, Aneeza Khairiyah Wan Hamizan

**Affiliations:** 1 Department of Otorhinolaryngology - Head and Neck Surgery, Universiti Kebangsaan Malaysia Medical Centre, Kuala Lumpur, MYS; 2 Department of Otolaryngology - Head and Neck Surgery, University of Malaysia Medical Centre, Kuala Lumpur, MYS

**Keywords:** conversion syndrome, sneezing, psychogenic, article review, intractable sneezing

## Abstract

Intractable sneezing is a diagnosis of exclusion and is mostly psychogenic. We reported a case of an 11-year-old girl who presented with uncontrollable bouts of sneezing for three weeks, which did not respond to conventional treatment. She was eventually diagnosed to have psychogenic intractable sneezing, which was triggered by an unfortunate family circumstance. She improved with psychotherapy and was discharged well. Literature review on intractable sneezing showed that patients were predominately female teenagers and mostly recovered after psychotherapy. Multidisciplinary team effort especially with a child psychiatrist is important for the treatment and follow-up of these patients. Imaging should also be routinely performed as some had underlying organic causes that presented as intractable sneezing.

## Introduction

Sneezing is a protective reflex to nasal irritation. It is mediated by a complicated two-step pathway consisting of nasal and respiratory phases and can be stimulated by various stimuli [1,2]. When the normal reflex pathway is disrupted or perturbed, sneezing can increase in severity and frequency to the extent of causing severe distress to the affected patients, leading to a diagnosis of intractable sneezing [3]. There are various etiologies of intractable sneezing, but the most commonly reported is the psychogenic cause [3]. We present a case of psychogenic intractable sneezing in an adolescent female who experienced debilitating sneezing following an unfortunate incident. Studies in the literature were reviewed and summarized from 1995 onward, with the following keywords: “intractable sneezing”, “psychogenic sneezing”, and “paroxysmal sneezing”.

## Case presentation

A healthy 11-year-old girl presented with uncontrollable bouts of sneezing for three weeks. This started after coming home from swimming and since then, she would have sudden episodes of fierce sneezing, as much as 10 to 15 times per minute, lasting for a few hours. There was no precipitating or relieving factor. There were no associated nasal symptoms, such as nasal itchiness, nasal blockage, nasal discharge, excessive lacrimation, or even cough. She had multiple visits to the general practitioner and was given intranasal steroidal sprays and even courses of antibiotics. The sneezing spell subsided for a few days and recurred in a more severe manner. It is interesting to note that the sneezing did not occur during her meal and sleeping time. It did, however, affect her daily activities, and her studies in school were greatly disturbed.

She was brought for an ENT consultation at a private hospital and underwent functional endoscopic sinus surgery, as minimal mucosal thickening on the left ethmoidal sinus was noted. However, the sneezing bouts continued at day 1 postoperatively and became more severe following that. She was then referred to our center for a second opinion.

During the consultation, the child was having one of her sneezing spells. She was sneezing with her eyes opened and it was not precipitated by deep drawing of breath. She denied any history of physical or emotional abuse. Nasal endoscopy revealed healthy nasal mucosa, normal inferior turbinate, and enlarged adenoids.

The skin prick test for allergies was negative, and a magnetic resonance imaging (MRI) scan of the brain revealed normal findings. She was eventually referred to pediatric and psychiatric teams for further assessment. Shockingly, after a few sessions with the child psychiatrist, she revealed that she was touched inappropriately by a close family member on a few occasions. She was mentally disturbed by the incidences. The diagnosis of psychogenic intractable sneezing was made. Her parents were informed of her condition, and she was commenced on psychotherapies. Her symptoms improved eventually, and her sneezing attacks ceased after the therapy sessions. Verbal consent was obtained from the patient and her parents. Her anonymity was preserved throughout the case report.

## Discussion

Sneezing was first defined by Brubaker in 1919 as spasmodic expiration preceded by one or more spasmodic inspirations [4]. A simple sneeze is initiated by a complicated two-step pathway. It starts with a sensory or nasal phase followed by an efferent or respiratory phase. The sensory phase is activated by chemical or mechanical stimulants onto the nasal mucosa. These sensory impulses travel via the ethmoidal and olfactory nerve to the sneezing center, which is located in the dorsolateral medulla [1,2]. This explains why patients with medulla stroke, lateral medullary syndrome, and sometimes neoplasm of this region may lose the ability to sneeze [5-7].

Mucosal edema and nasal secretions resulting from the nasal phase will then activate the second phase of sneezing, which is the respiratory phase. The respiratory phase requires synchronization of the respiratory, pharyngeal, and laryngeal musculature [8]. This efferent phase begins with eyes closure and an initial deep inspiration follows by forced expiration against a closed glottis causing increased intrathoracic pressure. With the opening of the glottis, the air is expelled explosively through the mouth and nose.

Intractable sneezing could be due to numerous etiologies, including allergic reactions, infections, anatomical abnormalities such as septal deviation, foreign body, and turbinate hypertrophy, seizure disorders, and vasomotor nasal congestion [9,10]. Interestingly, uncontrollable sneezing has also been reported as the sole presenting complaint in patients with medulla stroke [5,11]. However, the majority of reported cases of intractable sneezing are psychogenic. Thus, psychogenic intractable sneezing is defined as “sudden violent sneezing of unusual frequency and duration that is generally psychogenic in origin and is resistant to usual treatment” [3]. It was first reported in 1949, and over the years, most of the patients reported were adolescent girls aged 10 to 14 years [12-14]. It is a diagnosis of exclusion and a symptom of conversion disorder.

Articles search from PubMed and Google Scholar from the year 1995 and beyond using keywords “intractable sneezing”, “psychogenic sneezing”, and “paroxysmal sneezing” revealed 17 case reports that involved a total of 19 patients (Table 1). Among patients diagnosed with psychogenic sneezing, the age ranged from 8 to 16 years, and they were predominately females. Most of them seek treatment within two weeks of presentation, and their sneezing attacks did not occur during sleep. All reported patients received psychotherapy with or without anxiolytic medications. Two patients were even treated successfully with placebo. However, imaging studies were not routinely performed for all patients.

**Table 1:** Case Reports on Intractable Sneezing

	Author	Year	Age	Gender	Symptom Durations	Occur at Sleep	Imaging	Treatment	Etiology
1	Mathew et al. [15]	2017	10	Male	5 days	No	None	Psychotherapy/ alprazolam	Psychogenic
2	López-Chiriboga et al. [16]	2016	55	Female	-	-	Yes	Plasmapheresis	Neuromyelitis optica spectrum disorder
3	Mangot et al. [17]	2015	Teen	Female	15 days	No	None	Psychotherapy/ sertraline	Psychogenic
4	Mathis et al. [18]	2014	50	Male	3 weeks	No	Yes	Surgery	Papillary cardiac fibroelastoma
5	Then et al. [11]	2013	95	Male	1 day	-	Yes	-	Ischemic stroke
6	Chowdary [19]	2013	11	Female	2 months	-	Yes	Psychotherapy	Psychogenic
7	González-Díaz et al. [20]	2012	14	Female	4 days	No	None	Psychotherapy	Psychogenic
8	Sulemanji et al. [21]	2011	11	Female	14 days	No	Yes	Psychotherapy	Conversion disorder
9	Guner et al. [22]	2010	12	Female	1 day	No	Yes	Haloperidol	Psychogenic
10	Varshney et al. [23]	2010	32	Female	3 days	No	Yes	Psychotherapy/ alprazolam	Conversion disorder
11	Parke [24]	2009	7	Male	2 years (morning)	No	Yes	Surgery resection	Posterior fossa tumor
12	De Sousa [25]	2008	11	Female	7 days	No	Yes	Psychotherapy	Psychogenic
13	Bhatia et al. [26]	2004	16	Female	2 days	No	Yes	Psychotherapy/ alprazolam	Psychogenic
14	Bhatia et al. [26]	2004	16	Female	5 days	No	Yes	Psychotherapy	Psychogenic
15	Lin et al. [27]	2003	8	Female	8 days	No	None	Placebo	Psychogenic
16	Lin et al. [27]	2003	10	Female	3 days	No	None	Placebo	Psychogenic
17	Gopalan and Browning [9]	2002	12	Female	7 days	No	None	Psychotherapy	Psychogenic
18	García Callejo et al. [28]	2000	13	Male	18 days	No	Yes	Psychotherapy	Psychogenic
19	Goldfarb et al. [29]	1995	25	Female	3 months	-	Yes	Ethmoidectomy	Ethmoid sinusitis/iatrogenic

Detailed history-taking is essential in eliciting possible underlying stressors and the psychiatric features of the disorder. Pre-existing psychiatric diagnosis, psychosocial circumstances, history of previous conversion symptoms, or interpersonal relationship issues should be explored and addressed accordingly. Building good rapport with patients and their family is important, and the approach should be non-judgmental. In this case, the history of sexually inappropriate touching was only disclosed after multiple up-close encounters with a trustable child psychiatrist.

Physical examination for patients with psychogenic intractable sneezing is mostly unremarkable. Careful observation may be able to distinguish between physiology and psychogenic sneezing as they demonstrate numerous atypical characteristics. Patients tend to sneeze with their eyes open, associated with minimal inspiratory phase and minimal aerosolization of secretions, as well as minimal facial expression. The rate and rhythm of sneezing are abnormal, and they are resistant to conventional treatments [14].

Furthermore, investigations, such as blood tests, allergies tests, or even imaging, may also be normal in most cases. However, routine imaging is still advocated to rule out any underlying pathology. For instance, Parke reported a case of a seven-year-old boy who had intractable sneezing and was eventually diagnosed with a posterior fossa tumor [24]. Incidental findings on the imaging may also be the cause of the intractable sneezing, as evidenced in the case reported by Goldfarb et al., whereby a patient with intractable sneezing was found to have ethmoidal sinusitis on computed tomography (CT) imaging, which improved after endoscopic ethmoidectomy [29]. Imaging is also important especially for elderly patients with intractable sneezing as most of them would have an underlying pathology.

Several treatment modalities have been suggested for intractable sneezing in previous literature with varying success rates [14,22,27]. Medications such as intranasal corticosteroids, antihistamines, decongestants, subcutaneous epinephrine, and even anxiolytic medications have been used [22,27]. Placebo treatment with isotonic sodium chloride had also been used with satisfying results [27]. For psychogenic intractable sneezing, psychotherapy is the mainstay of treatment. Most of them recovered after psychotherapy. Family members’ participation and involvement may be helpful, especially in pediatric or adolescent patients. If this fails, hypnosis or even amytal interview can be used as a last resort to get additional information about the psychogenic stressor contributing to the symptom expression [14]. Patients with psychogenic intractable sneezing should be given long-term medical follow-ups as they may develop an organic or psychiatric illness later in their lives.

## Conclusions

Intractable sneezing is a debilitating condition and is commonly due to patients' underlying psychogenic illness. Detailed history taking and physical examination, as well as imaging, should be performed to rule out any underlying organic causes as summarized in the literature review. Clinical suspicion, sensible and empathic approach, and good family support would enable prompt diagnosis and treatment, which, in turn, would reduce future physical and psychosocial complications.
